# Effects of the Sludge Retention Time and Carbon Source on Polyhydroxyalkanoate-Storing Biomass Selection under Aerobic-Feast and Anoxic-Famine Conditions

**DOI:** 10.1021/acssuschemeng.1c02973

**Published:** 2021-07-08

**Authors:** Nicola Frison, Marco Andreolli, Alice Botturi, Silvia Lampis, Francesco Fatone

**Affiliations:** † Department of Biotechnology, University of Verona, Strada Le Grazie 15, 37134 Verona, Italy; ‡ Department of Science and Engineering of Materials, Environment and Urban Planning-SIMAU, Marche Polytechnic University, via Brecce Bianche 12, 60131 Ancona, Italy

**Keywords:** volatile fatty acids, mixed microbial culture, polyhydroxyalkanoate, polymerase chain reaction, microbial community analysis

## Abstract

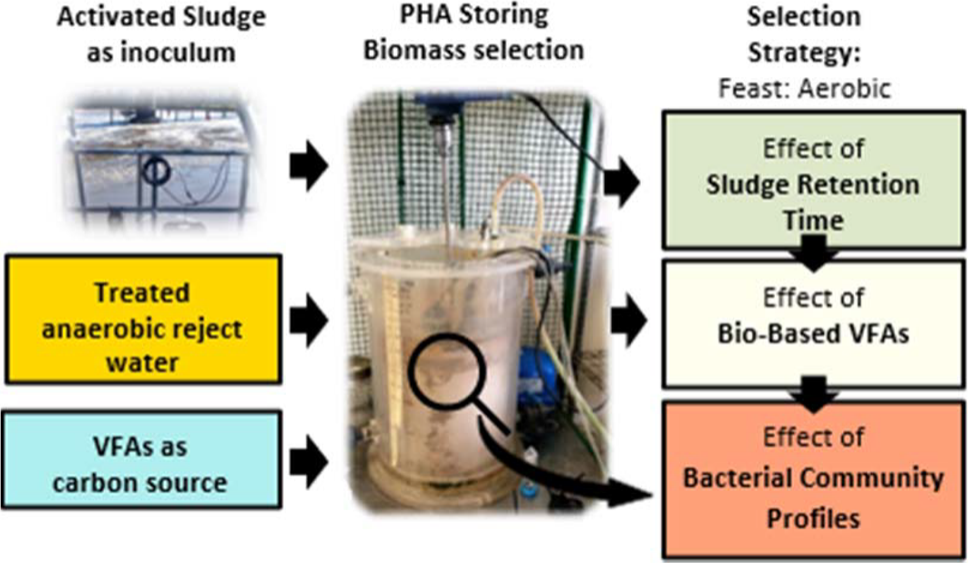

Polyhydroxyalkanoates (PHAs) are versatile biodegradable polymers produced by bacteria and are suitable for many downstream applications. They can be produced inexpensively from mixed microbial cultures under feast and famine conditions in the presence of biobased volatile fatty acids (VFAs). Here, we investigated the effect of changing the sludge retention time (SRT) and the addition of fermented cellulosic primary sludge (CPS) as a carbon source on the selection of PHA-storing biomass when applying the feast and famine strategy under aerobic and anoxic conditions, respectively. Increasing the SRT from 5 to 7–10 days enhanced PHA yields under feast conditions from 0.18 gCOD_PHA_/gCOD_VFA_ (period 1) to 0.40 gCOD_PHA_/gCOD_VFA_ (period 2). The use of fermented CPS as a carbon source (period 3) increased PHA yields to 0.62 gCOD_PHA_/gCOD_VFA_ despite the presence of biodegradable non-VFA fractions. Microbial characterization by denaturing gradient gel electrophoresis and fluorescence in situ hybridization revealed high microbial speciation during the three experimental periods. In period 3, the dominant genera were *Thauera*, *Paracoccus*, and *Azoarcus*, which accounted for ∼95% of the total microbial biomass.

## Abstract 2

The effect of sludge retention time and bio-based volatile fatty acids on the selection of polyhydroxyalkanoate-storing biomass.

## Introduction

Plastic materials are important aspects of our economy and society, but they threaten the environment and the health of humans and other animals. The EU produces ∼60 million tonnes of plastic waste per year from most economic sectors, but only 5 million tonnes is recycled and another 25 million tonnes is lost, including misplaced waste and process losses during recycling.^1^ Biobased plastics account for only 0.4% of the total, but this niche sector nevertheless offers an opportunity for further growth.^1,2^ This sector includes the polyhydroxyalkanoates (PHAs), which are biodegradable polymers produced and stored by various bacteria as cytoplasmic inclusion bodies that function as energy reserves during periods of carbon starvation.^3,4^ These versatile products are suitable for multiple applications and degrade naturally in the environment, increasing their market potential.^5^

The EU currently produces ∼2000 tonnes of PHAs per year, mainly using pure bacterial strains growing on expensive carbon sources such as glucose, resulting in a market price of 4–9 €/kg, roughly six times more expensive than standard petrochemical plastics.^6,7^ However, PHAs can also be produced from selected mixed microbial cultures (MMCs) under feast and famine conditions in the presence of biobased volatile fatty acids (VFAs) as low-cost building blocks obtained from the acidogenic fermentation of organic waste, sewage sludge, and wastewater.^8−11^ In this scenario, waste and wastewater treatment plants could become sustainable biorefineries that deliver new products and resources recovered from waste streams.^12^ For example, municipal wastewater contains a significant quantity of toilet paper that could be sieved and recovered as cellulosic primary sludge (CPS) for fermentation to obtain VFAs as PHA precursors.^13−15^

A productivity of 1.0–1.2 kg PHA per capita per year was recently validated using MMC-derived CPS as an external carbon source under aerobic-feast and anoxic-famine conditions as a selection strategy.^16,17^ Furthermore, the aerobic-feast and anoxic-famine conditions were investigated for the selection of PHA-storing biomass and nitrogen removal via nitrite in the main wastewater treatment line to meet water effluent quality goals in terms of chemical oxygen demand (COD), nitrogen (N), and phosphorus (P).^18^ However, the scale-up of PHA production requires a clear understanding of the best design parameters to maintain bioprocess performance over a long duration.

Most investigations involving the feast and famine strategy under complete aerobic conditions have focused on the effect of the sludge retention time (SRT) on biomass selection, but the results have been variable. Shorter SRTs have often been shown to increase PHA productivity,^19,20^ but other authors found that faster-growing organisms accumulate less polyhydroxybutyrate.^21,22^ Activated sludge processes with a short SRT (3 days) may select for microbial communities with a higher PHA production capacity than a longer SRT (10 days).^19^ Under anoxic conditions, an increase in the organic loading rate reduces the PHA storage capability.^23^ However, the effect of the SRT and carbon source in terms of PHA production and nitrogen removal efficiency has not been investigated when the selection strategy is based on aerobic-feast and anoxic-famine conditions. Moreover, to the best of our knowledge, this is the first study on comparing the effect of CPS fermentation and synthetic VFAs as carbon sources on PHA-storing biomass selection. We evaluated changes in the community of PHA-storing bacteria over time using two culture-independent techniques: fluorescence in situ hybridization (FISH) and the polymerase chain reaction with denaturing gradient gel electrophoresis (PCR-DGGE). The overall goal was to identify a link between the applied operating conditions and the abundance of microbial populations involved in PHA production.

## Materials and Methods

### Configuration of the Process Units

**Figure 1:**
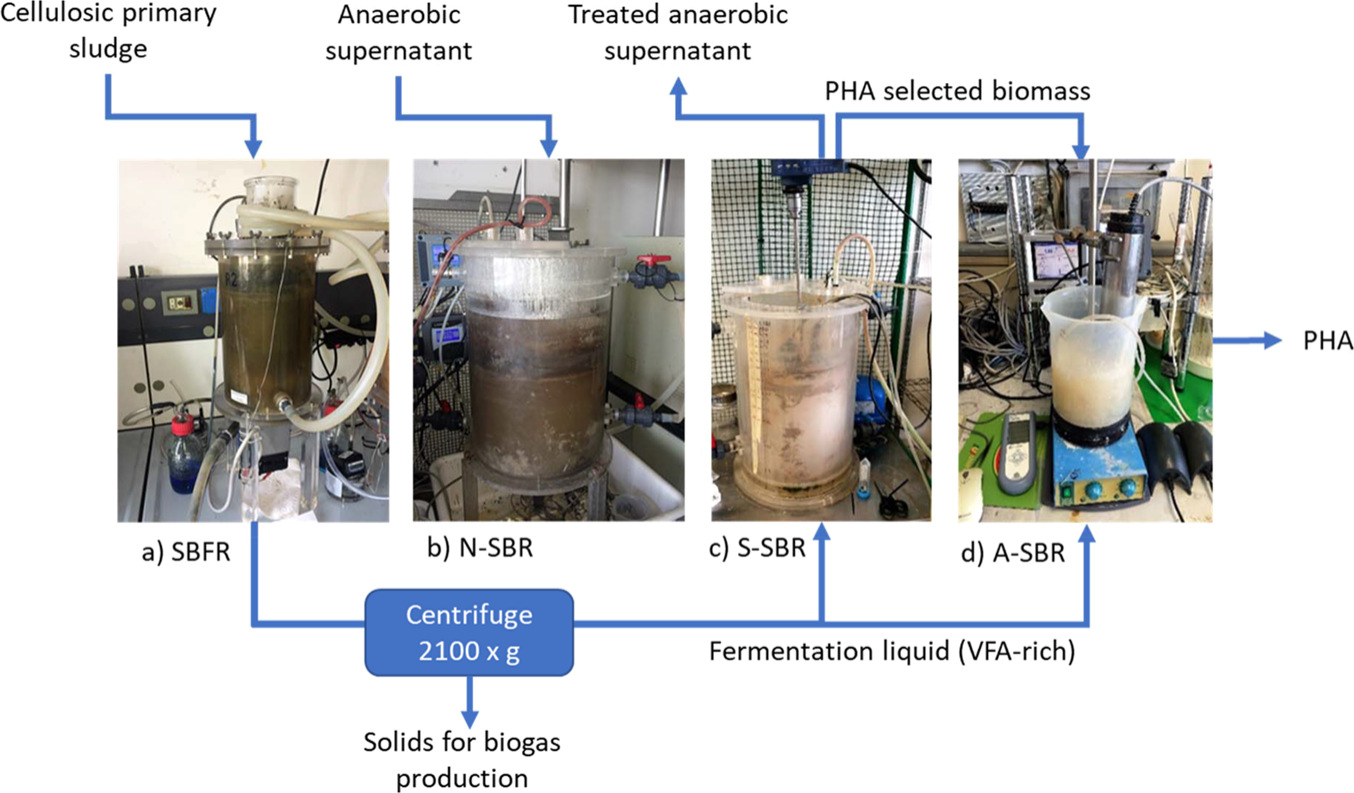
Overall laboratory-scale configuration of the sequencing batch reactor (SBR). (a) Sequencing batch fermentation reactor (SBFR). (b) Nitritation sequencing batch reactor (N-SBR). (c) Selection sequencing batch reactor (S-SBR). (d) Accumulation sequencing batch reactor (A-SBR).

The effects of the SRT and carbon source on the selection of PHA-storing biomass under aerobic-feast and anoxic-famine conditions were investigated in a sequencing batch reactor (SBR) with a 28 L working volume.^17^ The SBR was used for the selection of PHA-storing biomass and nitrogen removal from the anaerobic reject water based on the configurations already described.^16^ The overall laboratory-scale configuration is shown in Figure 1, although we were primarily concerned with the operation of the selection SBR (S-SBR). The configuration is described in detail in the Supporting Information.

During the aerobic feast, the oxygen was provided by two air diffusers connected using a Tetratec APS 300 volumetric air blower (37 W) with a maximum flow rate of 40 L/min (Tetra, Melle, Germany). The aeration was sufficient to achieve a maximum oxygen concentration of 8 mg/L at 25 °C. The oxygen concentration in the mixed liquor was monitored using an oxygen sensor (Hach-Lange, Düsseldorf, Germany), and the mixed liquor was continually agitated under aerobic and anoxic conditions using an RW 20 overhead stirrer (IKA-Werke, Staufen, Germany). The feeding and discharge of the S-SBR were achieved using peristaltic pumps. The electromechanical components were controlled using a programmable logic controller. The S-SBR was inoculated with activated sludge from the oxidation tank of the municipal wastewater treatment plant in Carbonera (Italy).

The specific activities of the inoculum in terms of the maximum oxygen uptake rate (sOUR), nitrification rate (ammonia uptake rate, sAUR), and denitrification rate (nitrogen utilization rate, sNUR) were estimated as previously described^24^ (Table S1).

The selection of PHA-storing biomass was achieved by adding VFAs during the aerobic-feast phase with a volumetric organic loading rate (vOLR) of ∼1.30 kgCOD(VFA)/m^3^ day and N-reject water during the anoxic-famine phase based on a volumetric nitrogen loading rate (vNLR) of 0.55 kgN/m^3^ day. The VFAs used to establish the aerobic-feast conditions were a mixture of synthetic acetic and propionic acids or were produced from fermented CPS in a sequencing batch fermentation reactor (SBFR). The anoxic-famine conditions in the S-SBR were established by switching off the blowers and feeding ∼120 mg/L per cycle of nitrite as an electron acceptor. The nitrite was produced in a nitritation SBR (N-SBR) treating reject water from the anaerobic digestion of sewage sludge (Table S2).^16,17^ This strategy allowed nitrite removal from the anaerobic reject water and the growth of biomass driven by the consumption of PHAs as a carbon source. The chemical and physical characteristics of the fermentation liquid from the CPS and anaerobic reject water fed to the S-SBR are summarized in Table 1.

**Table 1:** Chemical and Physical Characteristics of the CPS Fermentation Liquid and the Effluent from the N-SBR (n.a. = Not Available; n.d. = Not Detected)

parameter	unit	effluent from N-SBR	CPS fermentation liquid
soluble COD^a^	mgCOD/L	32 ± 2	9405 ± 223
NO_2_-N^b^	mgN	882 ± 76	n.d.
NH_4_-N^c^	mgN/L	115 ± 7	n.d.
HAc^d^	mgCOD/L	n.a.	4255 ± 852
HPr^e^	mgCOD/L	n.a.	2003 ± 169
HBut^f^	mgCOD/L	n.a.	984 ± 775
total VFAs^g^	mgCOD/L	n.a.	7242 ± 1139

a Soluble COD: soluble chemical oxygen demand.

b NO_2_-N: nitrite as nitrogen.

c NH4-N: ammonium as nitrogen.

d HAc: acetic acid.

e HPr: proprionic acid.

f HBut: butyric acid.

g Total VFAs: total volatile fatty acids.

On each day, part of the selected biomass in the S-SBR was harvested as shown in Table 2 and used as the inoculum for an accumulation SBR (A-SBR) to evaluate the maximum PHA concentration under sequential spikes of VFAs. The operating conditions of the A-SBR are described in the Supporting Information.

**Table 2:** Operating Parameters of the S-SBR during the Three Experimental Periods

parameter	period 1 (days 0–39)	period 2 (days 40–106)	period 3 (days 107–145)	previous study^16^	previous study^17^
type of carbon source	synthetic VFAs	synthetic VFAs	VFAs from CPS	VFAs from CPS	VFAs from mixed sludge
temperature					
vNLR (kgN/m^3^ day)	0.55	0.48 ± 0.19	0.56 ± 0.02	0.49 ± 0.11	0.50 ± 0.11
vOLR (kgCOD/m^3^ day)	1.32 ± 0.12	1.32 ± 0.12	1.18 ± 0.32	1.58 ± 0.10	1.39 ± 0.11
SRT (day)	5	7–10	7–10	6–7	12 ± 3

### Operating Conditions of the S-SBR

The experimental activity lasted 145 days with three main periods, during which the applied SRT was changed from 5 days (period 1, days 0–39) to 7–10 days (period 2, days 40–106; period 3, days 107–145). All experimental periods lasted more than three times longer than the SRT of the biomass to ensure that the results were significant. Furthermore, in periods 1 and 2, the same synthetic mixture of VFAs was used as a carbon source, allowing us to evaluate the effect of the SRT alone. The carbon source comprised ∼10 gCOD(VFAs)/L with a 70:30 ratio of acetic and propionic acid, reflecting the typical composition of VFAs produced by the acidogenic fermentation of CPS.^14^ During period 3 (days 107–145), the synthetic mixture of VFAs was replaced with the CPS fermentation liquid to solely evaluate the effect of the carbon source on the selection of PHA-storing biomass. Other operating parameters, such as the vOLR and vNLR, were maintained almost constant during the experimental periods as previously reported.^16^ The operating parameters of the S-SBR during the three periods are summarized in Table 2.

**Figure 2:**
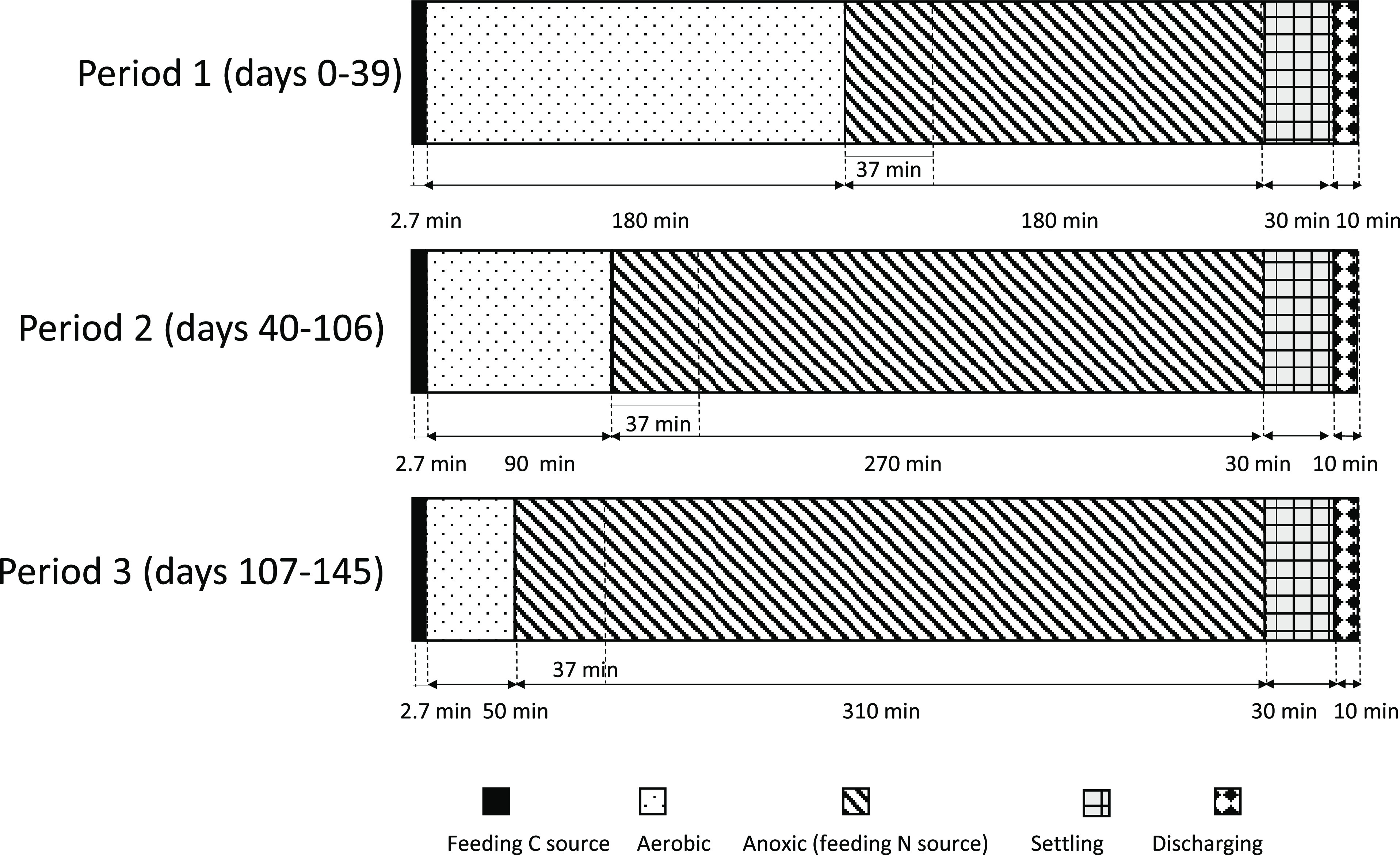
Cycle configuration of the selection sequencing batch reactor (S-SBR) during each experimental period.

Further details of the cycle configurations are shown in Figure 2.

### Calculations

The concentration of VFAs represented the sum of all C-2 to C-6 acids expressed as COD, as shown in eq 1:
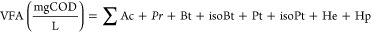
1where Ac is acetate, Pr is propionate, Bt is butyrate, isoBt is iso-butyrate, Pt is pentanaoate, isoPt is iso-pentanaoate, He is hexanoate, and Hp is heptanoate.

The F/F ratio is the length of the feast phase divided by the length of the famine phase, as shown in eq 2:
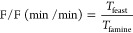
2where *T*_feast_ is the time required for the complete uptake of VFAs and *T*_famine_ is the period (under aerobic and/or anoxic conditions) of the cycle between the complete depletion of the VFAs and the end of the cycle.

The relative fraction of PHA in the biomass was calculated using eq 3:
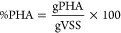
3where gPHA is the dry mass of PHA determined after the extraction and gVSS is the total dry mass of volatile suspended solids. The specific VFA uptake rate −qVFA (mgCOD/gMLVSS h) and the PHA storage rate qPHA (mgPHA/gMLVSS h) were determined by linear regression analysis by plotting the concentration of VFAs and PHA as a function of time. The results were normalized against the concentration of mixed liquor volatile suspended solids (MLVSS).

The concentration Xa of the S-SBR was calculated by subtracting the concentration of PHA (g/L) from the concentration of VSS (g/L). Xa was then converted into COD by a stoichiometric value of 1.42 gCOD/gXa as previously reported.^25^

The VFA utilization rate −qVFA (mgCOD/gXa h) was calculated by dividing the concentration of consumed VFAs by the duration of the feast phase. The PHA storage yield *Y*_PHA/VFA_ (gCOD_PHA_/gCOD_VFA_) and growth yield *Y*_X/VFA_ (gXa/gCOD_VFA_) during each experimental period were determined using eqs 4 and 5:
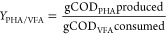
4
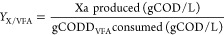
5

### Analytical Methods

The systems were monitored by sampling the influent and effluent as well as taking samples during the cycle of the S-SBR in order to evaluate the VFA uptake rate and the PHA storage rate of the biomass. The concentration of the mixed liquor suspended solids (MLSS), MLVSS, COD, soluble COD (sCOD), total Kjeldahl nitrogen (TKN), ammonium (NH_4_-N), and total phosphorus (TP) were determined as previously reported.^26^ Nitrite (NO_2_-N), nitrate (NO_3_-N), and phosphate (PO_4_-P) concentrations were determined using a Dionex ICS-900 ion chromatograph and AS14 column (Thermo Fisher Scientific, Waltham, MA, USA). The VFA content was determined by liquid chromatography using a Dionex ICS-1100 with AMMS ICE 300 as a suppressor and AS23 as a separation column (Thermo Fisher Scientific). The PHA content was determined gravimetrically as previously described.^27^ The sludge volume index (SVI) was calculated by dividing the sludge volume after 30 min of settling in the Imhoff cone (mL/L) by the MLSS (g/L).

### PCR-DGGE Analysis, Sequencing, and Statistical Analysis

Microbial species were detected by polymerase chain reaction denaturing gradient gel electrophoresis (PCR-DGGE) on seven duplicate samples of sludge collected from the S-SBR after 0, 22, 48, 68, 107, 113, and 119 days. We also sampled the liquid fraction of the fermenter or the anaerobic supernatant. Samples were stored at −20 °C. Total DNA was extracted using the MP Biomedicals FastDNA Spin Kit for Soil (Thermo Fisher Scientific). PCR-DGGE analysis targeted the 16S rRNA V3 hypervariable region using a 30–60% denaturing gradient as previously described.^28^ DNA in the excised DGGE bands was re-amplified using non-GC-clamped primers p1-p2 and transferred to the pGEM-T vector (Promega, Milan, Italy) for transformation of *Escherichia coli* XL1-Blue competent cells (Agilent Technologies, Santa Clara, CA, USA).^29^ Inserts were sequenced by GATC Biotech (Cologne, Germany) and used as BlastN queries against the NCBI and EzBioCloud databases.^30,31^ Sequences obtained by PCR-DGGE were deposited with accession numbers MW776615–MW776626. The similarity indexes among DGGE profiles were determined by UPGMA cluster analysis, and the dendrogram was constructed using UVIbandmap software (UVITEC, Cambrige, UK).

### FISH Analysis

Fluorescence in situ hybridization (FISH) was carried out on biomass from S-SBR sludge collected at the beginning of the experiment (period 1) and after 68 (period 2) and 119 (period 3) days. We fixed ∼0.5 mL of biomass in 1.5 mL of 4% paraformaldehyde (PFA), and FISH was carried out as previously described^32^ using Cy3-labeled probe THAU646 specific for the genus *Thauera*, Cy3-labeled probe PAR651 specific for the genus *Paracoccus*, and Cy3-labeled probe AZA645 that recognizes most members of the *Azoarcus* cluster.^33−35^ Samples were counterstained with 4′,6-diamidino-2-phenylindole (DAPI), which detects most bacteria. The hybridized samples were viewed under a DM2500 upright fluorescence microscope with 40× magnification for image capture (Leica Microsystems, Wetzlar, Germany). Forty random images from each sample were analyzed using ImageJ software. The abundance of the diverse groups was expressed as a percentage of all bacteria (area occupied by probe-binding cells), and statistical analysis was carried out as previously described*.*^36^

## Results and Discussion

### Effect of the SRT on the Performance of the S-SBR

Although the storage and degradation of PHA involves the same metabolic processes under both anoxic and aerobic conditions, the storage and growth yields change because the availability of ATP varies in the presence of different electron acceptors.^6,23^ Under aerobic conditions, a storage yield of 0.85 gCOD_X,STO_/gCOD of substrate consumed was reported, together with a growth yield of 0.63 gCOD_Xa_/gCOD_X,STO_.^37^ In contrast, under complete anoxic conditions, the storage yield was 0.80 gCOD_X,STO_/gCOD of substrate consumed and the growth yield was 0.54 gCOD_Xa_/gCOD_X,STO_, representing differences of −6 and −14%, respectively, compared to aerobic conditions.^37^ This means that, in addition to the SRT, the biomass achieves different growth yields depending on the type of environment selected for the feast and famine conditions. Multiplying the aerobic storage yield (0.85 gCOD_X,STO_/gCOD of substrate consumed) by the anoxic growth yield (0.54 gCOD_Xa_/gCOD_X,STO_) of stored compounds amounts to 0.46 gCOD_Xa_/gCOD of substrate consumed, which can be considered as the maximum biomass growth yield achievable by the combination of aerobic-feast (storage) and anoxic-famine (growth) conditions.

**Figure 3:**
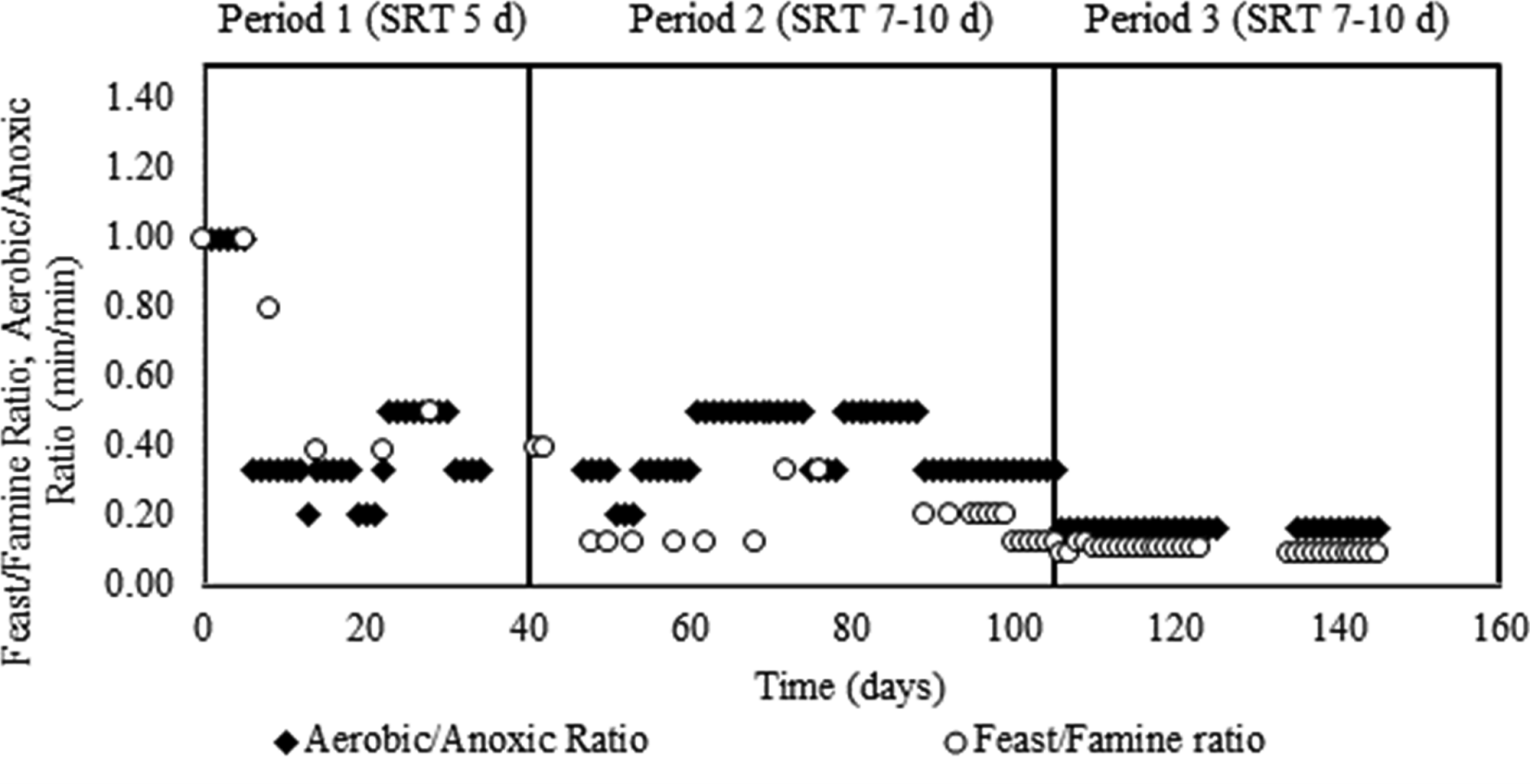
Relationship between the F/F ratio and the aerobic/anoxic ratio.

**Figure 4:**
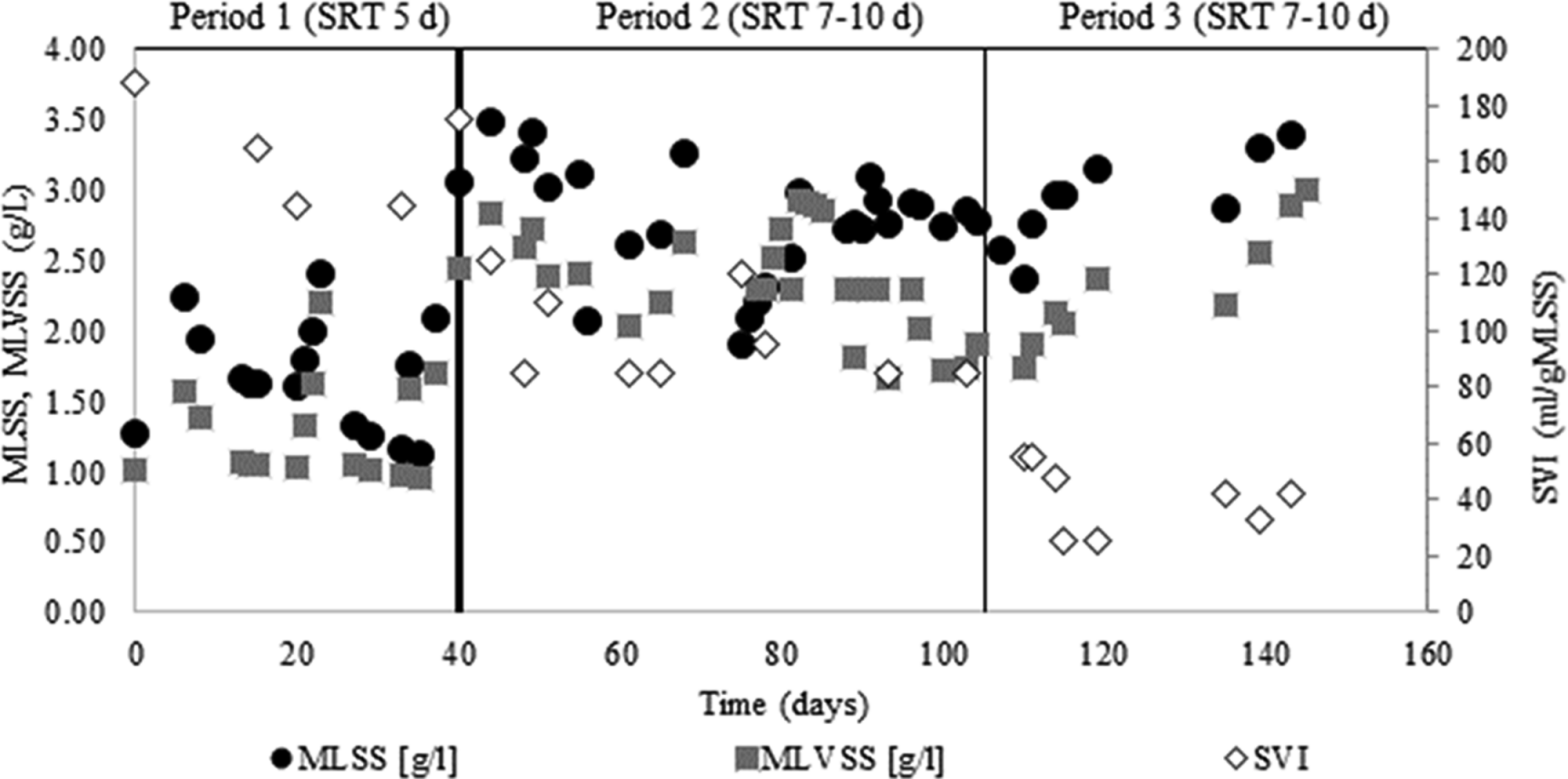
Profile of the biomass (MLSS and MLVSS) and the SVI during the three experimental periods.

During the first 14 days, the observed F/F ratio fell from 1 min/min (days 0–2) to 0.38 min/min (days 14–41), revealing an increase in the VFAs uptake rate (Figure 3). The F/F ratio was similar to the aerobic/anoxic ratio, which means that the anoxic conditions began as soon as the VFAs were taken up under aerobic conditions. The raid decrease in the F/F ratio could reflect the constant loss of biomass observed in the effluent, resulting in a variable MLSS concentration of 1.12–2.4 g/L in the S-SBR (Figure 4). Biomass depletion during the PHA biomass selection may be due to the presence of bacteria with poor PHA-storage capacity in the initial inoculum.^38^ However, the average concentration of MLSS during this period was 1.68 g/L, ∼80% of which was the volatile fraction (MLVSS). The shortest SRT applied in combination with the length of the aerobic and anoxic phases resulted in the highest observed *Y*_X/VFA_ of 0.37 gCOD_Xa_/gCOD consumed. This was the highest value achieved in our experiments but was ∼20% lower than the maximum achievable value. Under these conditions, the food/microorganism ratio (F/M) was 1.16 ± 0.25 gCOD_VFA_/gXa (Table 3), which is more than double the value reported at the same SRT under complete aerobic F/F conditions.^38^

**Table 3:** S-SBR Performance during Periods 1, 2, and 3

parameters	unit	period 1 (days 0–39)	period 2 (days 40–106)	period 3 (days 107–145)	previous study^16^	previous study^17^
–qVFA^feast,a^	gCOD_VFA_/gXa h	173 ± 18	228 ± 22	282 ± 26	289–322	239 ± 7
qPHA^feast,b^	gPHA/gXa h	31 ± 4	91 ± 3	176 ± 14	184–231	89 ± 7
F/M^c^	gCOD_VFA_/gXa	1.16 ± 0.25	0.67 ± 0.19	0.63 ± 0.09	0.49–0.57	0.37 ± 0.07
SRT^d^	day	5	7–10	7–10	6–7	12 ± 3
*Y*_X/VFA_^e^	gCOD_Xa_/gCOD_VFA_	0.37 ± 0.02	0.31 ± 0.02	0.35 ± 0.03	0.41–0.44	0.42
feast *Y*_PHA/VFA_^f^	gCOD_PHA_/gCOD_VFA_	0.18 ± 0.01	0.40 ± 0.05	0.62 ± 0.04	0.64–0.74	
%PHA (end of feast)^g^	% (gPHA/gVSS)	19.0	9.1	9.7	10%	6%
%PHA (end of famine)^h^	% (gPHA/gVSS)	16.4	3.8	5.5	0.3%	0.6%

a –qVFA^feast^: specific volatile fatty acids uptake rate under feast conditions.

b qPHA^feast^: specific PHA production rate under feast conditions.

c F/M: food/microorganisms ratio.

d SRT: solid retention time.

e *Y*_X/VFA_: yield of active biomass based on VFAs consumed.

f Feast *Y*_PHA/VFA_: yield of PHA produced based on VFAs consumed under feast conditions.

g %PHA (end of feast): percentage of PHA based on volatile suspended solids at the end of feast conditions.

h %PHA (end of famine): percentage of PHA based on volatile suspended solids at the end of famine conditions.

The same study reported a stable selection process in which the biomass settled efficiently (SVI < 100 g/mL) operating with an F/M ratio of 0.69 gCOD_VFA_/gXa (Table 3).^38^ In period 1, the presence of relatively high levels of nitrite combined with nondegraded storage compounds in the biomass (∼16.4%) triggered uncontrolled denitrification in the S-SBR, which caused swelling and poor settling of the biomass, resulting in an SVI profile that remained higher than 140 mL/gMLSS (Figure 4). In period 1, it is notable that poor settling reduced the actual SRT below the set value (5 days), which affected the concentration of biomass retained in the S-SBR. The depletion of the MLSS and MLVSS also affected the observed nitrite removal rate under anoxic conditions. Because the length of the S-SBR cycle was fixed at 360 min, the length of the anoxic period after the feast phase did not exceed 220 min, which limited the denitrification efficiency to an average of 28% and led to a high nitrite concentration in the effluent (294–554 mgN/L).

**Figure 5:**
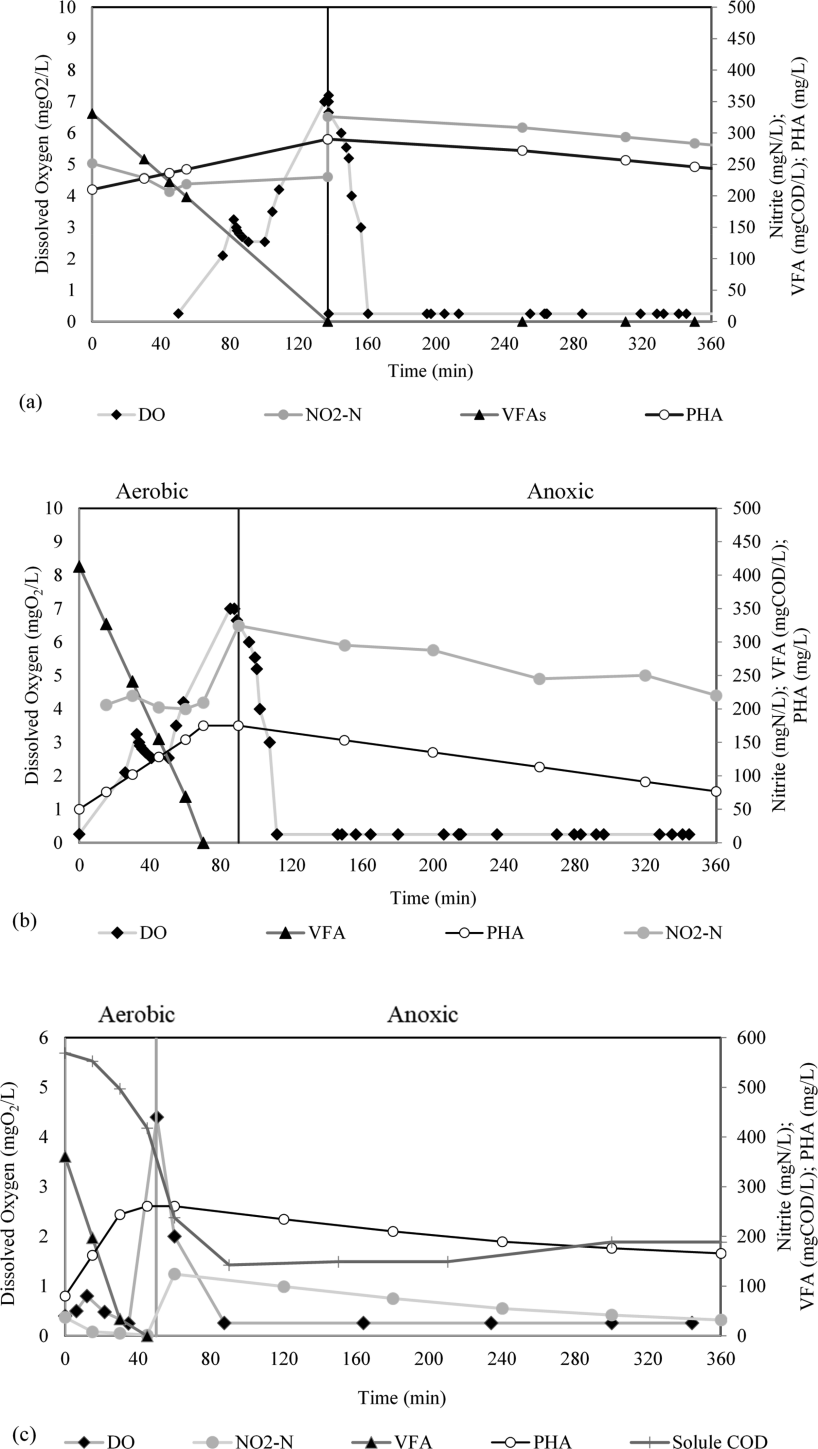
Typical profiles of electron acceptors (dissolved oxygen and nitrite), VFAs, and PHAs during the feast and famine phases of (a) period 1, (b) period 2, and (c) period 3.

The specific denitrification rates at 20 °C during periods 1 and 2 were very similar: 10.5 and 9.5 mgN/gVSS h, given SRTs of 5 and 7–10 days, respectively (Supporting Information). These denitrification rates were similar to those reported in an earlier study^17^ and could be linked to the nitrite removal rate in the presence of PHAs as the sole carbon source. During period 2, increasing the SRT to 7–10 days led to a slight decrease in *Y*_X/VFA_ to 0.31 gCOD_Xa_/gCOD consumed, which was 33% lower than the maximum achievable value. The biomass concentration in the S-SBR increased to 2.1 gMLVSS/L and the F/M ratio fell to an average of 0.67 ± 0.19 gCOD/gMLVSS. The F/M ratio in period 2 agreed with an F/M ratio of 0.49–0.57 gCOD/gMLVSS previously reported for an SRT of ∼7 days.^16^ The lower F/M ratio was also accompanied by a decrease in the F/F ratio to 0.13–0.33 min/min, allowing a relatively longer anoxic-famine phase. Compared with period 1, the lower organic load applied to the biomass favored the consumption of the stored PHAs under the anoxic-famine conditions driven by the denitrification process (Figure 5a,b). During period 2, the PHA concentration at the end of the feast phase was 9.1% (174 mgPHA/L), which decreased to ∼3.8% (76 mgPHA/L), resulting in a nitrite removal efficiency of ∼61%. The enhanced PHA degradation during the famine phase also had a positive effect on the SVI, which fell below 100 mL/gMLSS.

Based on these results, when the selection of PHA-storing biomass during the aerobic-feast and anoxic-famine phases is achieved under aerobic and anoxic conditions, the SRT affected the following mechanisms: (1) a shorter SRT led to a lower biomass concentration in the S-SBR and a longer feast phase was required; (2) accordingly, a shorter famine phase reduced the time available for the degradation of storage compounds and the denitrification efficiency was negatively affected; and (3) uncontrolled denitrification occurred during the settling phase due to the high nitrite concentration and residual PHA stored in the biomass.

### Effect of the Carbon Source

The effect of the carbon source was investigated by comparing periods 2 and 3, where the synthetic mixture of VFAs was replaced with the CPS fermentation liquid to reproduce the same environmental conditions reported in a previous study*.*^16^ The VFAs were consumed at an almost constant rate during periods 1 and 2, but this increased slightly during period 3, as shown by the VFA uptake rate −qVFA (282 ± 26 mgCOD/gVSS h). Although the OLR_VFA_ remained almost constant during each experimental period, the total OLR in period 3 was slightly higher due to the presence of the non-VFA fraction in the soluble COD of the fermentation liquid. During complete aerobic feast and famine phases, the biodegradable fractions of non-VFAs contained in the carbon source may hinder the enrichment of PHA-storing biomass. In one previous study, a shortened famine phase and poor enrichment of PHA biomass were caused by the presence of biodegradable non-VFA fractions of fermented molasses in the selection SBR.^39^ In another study, poor selective pressure on the PHA-storing biomass occurred despite a satisfactory F/F ratio (19–20%).^40^ Here, we found that the consumption of biodegradable non-VFA fractions was not favored during the anoxic-famine period, limiting the growth of non-PHA storing bacteria and leading to higher selectivity.^16,17,37^ This advantage makes the aerobic-feast and anoxic-famine strategy most effective when the biomass is fed with a complex carbon source, such as the VFAs produced from the fermentation of raw substrates.

During periods 1 and 2, the COD removal efficiency was 99%, indicating that almost all VFAs were utilized during the feast phase. In contrast, the COD removal efficiency during period 3 declined to 79%, although the VFAs were taken up completely during the feast phase. The residual soluble COD in the effluent of the S-SBR can be attributed to the non-VFA fraction, which was not completely degraded during the anoxic-famine phase. The ratio between the soluble COD and VFA concentrations of the CPS fermentation liquid was ∼77% (Table 1). As shown in Figure 5c, the soluble COD gradually decreased during the feast phase together with the depletion of the VFAs. At the end of the feast phase, the soluble COD concentration was ∼250 mgCOD/L, whereas the concentration of PHA increased to 9.7% (260 mgPHA/L).

When the famine phase started, the soluble COD dropped to 144 mgCOD/L at 90 min and remained stable up to 200 min before increasing slightly to 188 mgCOD/L at the end of the cycle due to the degradation of hydrolysable organic compounds in the mixed liquor. However, these residual organics did not affect the selective pressure, leading to the lowest F/F ratio (0.10 ± 0.01) and the highest *Y*_PHA/VFA_ (0.62 gCOD_PHA_/gCOD_VFA_) compared to periods 1 and 2 (Table 3). This promoted higher nitrite removal efficiency (86.2 ± 4.8%) due to the longer anoxic-famine phase, which favored the utilization of the stored PHAs as a carbon source for denitrification, in combination with the utilization of the biodegradable non-VFA compounds. During period 3, at the end of the famine phase, the concentration of PHA decreased to 5.5% (165 mgPHA/L). On the other hand, the presence of nutrients and a wider range of VFAs contributed to the biomass performance, confirming that CPS is a feedstock more prone to microbial degradation than synthetic VFAs without compromising the selected microbial community.

### PCR-DGGE Analysis

**Figure 6:**
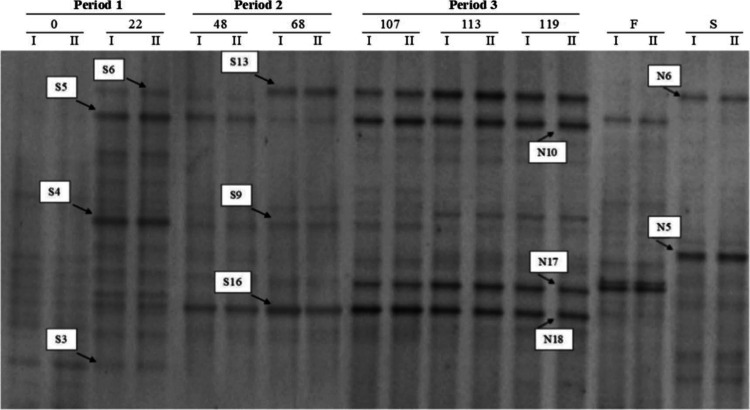
PCR-DGGE analysis of the S-SBR reactor. Bands represent the initial activated sludge (0) and the reactor contents after 22 (period 1), 48, 68 (period 2), 107, 113, and 119 (period 3) days from the beginning of the experiment. The fermentation liquid (F) and anaerobic supernatant (S) were analyzed in period 3. Arrows indicate bands excised from the gel for sequencing.

The structure of the microbial community was monitored by PCR-DGGE at different sampling time points (Figure 6). The DGGE profiles representing the inoculum sludge (sample 0) featured a large number of bands, but after 22 days, some well-defined dominant bands had emerged, which were excised from the gel for sequencing.

Sequencing and phylogenetic analysis (Table S3) revealed that the excised bands in period 1 represented the genera *Pseudomons* (S4 and S5) and *Thauera* (S3) as well as a poorly defined bacterial genus (S6). *Pseudomonas* species have already been found in batch systems for PHA production,^38,41^ and *Thauera* species have been detected in SBRs with both short SRTs (1–2 days) and long SRTs (10 days).^42−45^ The bacterial community had changed in period 2, suggesting that the different SRTs had a significant impact on microbial speciation. The excised bands in period 2 represented an unknown strain of α-Proteobacteria (S9) and the genera *Paracoccus* (S13) and *Thauera* (S16). Strains belonging to both of these genera are well-known PHA producers and are often detected among the most abundant PHA-storing bacteria in S-SBRs fed with VFAs under F/F conditions.^44,46−51^ In period 3, both the liquid fraction of the fermenter (F) and the anaerobic supernatant (S) were supplied as carbon sources to the S-SBR, and many bands were observed in both cases. The main supernatant bands were sequenced and were found to represent the classes Firmicutes (N5) and α-Proteobacteria (N6). Although different carbon sources were used, period 3 shared some bands with period 2 (e.g., S13 and S16). Sequencing analysis showed that the other dominant bands represented the genus *Thauera* (N17 and N18) and the class Flavobacteriia (N10). Although this class of bacteria was previously found in an S-SBR, its ability to store PHA was only reported once.^50−52^ Bacteria that do not store PHAs can therefore thrive and survive in an S-SBR.

**Figure 7:**
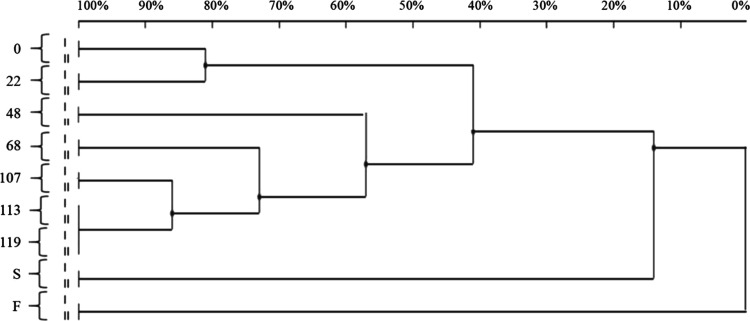
Dendrogram indicating the similarity indices of the different DGGE profiles based on samples collected from activated sludge (0) and after 22 (period 1), 48, 68 (period 2), 107, 113, and 119 (period 3) days from the beginning of the experiment. Both fermentation liquid (F) and anaerobic supernatant (S) were included in period 3.

The statistical analysis of DGGE banding was visualized in a dendrogram (Figure 7). Samples 0 and 22 showed ∼80% similarity. The most remarkable change in the bacterial community occurred when shifting from an SRT of 5 days (period 1) to 7–10 days (period 2), which reduced the similarity index to only 40% (comparison of sample 22 to samples 48, 68, 107, 113, and 119). As previously reported, the SRT applies strong selective pressure to PHA-storing bacteria.^8,48,51^ A short SRT may be responsible of the rapid utilization of the substrate for growth rather than polymer accumulation.^50^ For example, some genera such as *Amiricoccus* and *Azoarcus* are favored by an SRT of 10 days, whereas *Plasticicumulans* has often been detected in processes with short SRTs.^20,53^ The dendrogram revealed that the similarity index gradually increased during periods 2 and 3, with 58, 72, 85, and 100% similarities observed after 48, 68, 107, and 113 days respectively. Very low similarity indices (less than 10%) were observed between the S and F samples and samples collected during period 3. These results suggest that the operational parameters of the S-SBR were the main factors affecting bacterial speciation. In contrast, the different inputs used in period 2 (synthetic VFAs) and period 3 (fermentation liquid) appear to have a negligible impact on the selection of PHA-storing biomass.

### FISH Analysis

The DGGE data and similarity indices did not provide any information regarding bacterial abundance. Therefore, FISH analysis and the further statistical evaluation were carried out to quantify the main PHA-storing bacterial genera (*Thauera*, *Paracoccus*, and *Azoarcus*) in the S-SBR (Figure S1).^44,49^ The relative abundances of *Paracoccus* were 1.5 ± 0.02, 5.3 ± 0.03, and 19.1 ± 5.2% as a proportion of total bacteria in periods 1, 2, and 3 respectively. Moreover, the relative abundances of *Azoarcus* were 1.2 ± 0.01% in period 1, 3.2 ± 0.03% in period 2, and 17.4 ± 4.9% in period 3. Therefore, although bands representing *Azoarcus* were not identified by PCR-DGGE analysis, this genus was clearly present in the S-SBR. Eventually, the relative abundance of *Thauera* increased from 3.0 ± 0.02% in period 1 to 17.4 ± 4.4% in period 2 and 58.2 ± 11.1% in period 3. *Thauera* is therefore the most abundant genus. Taken together, these three genera represented 5.7 ± 0.04, 25.9 ± 4, and 94.7 ± 13.2% of the total bacterial population in periods 1, 2, and 3, respectively (Table 4).

**Table 4:** Relative Abundance of the Genera *Thauera*, *Paracoccus*, and *Azoarcus* in the S-SBR during Periods 1, 2, and 3

genus	period 1	period 2	period 3
*Thauera* (%)	3.0 ± 0.02	17.4 ± 4.4	58.2 ± 11.1
*Paracoccus* (%)	1.5 ± 0.02	5.3 ± 0.03	19.1 ± 5.2
*Azoarcus* (%)	1.2 ± 0.01	3.2 ± 0.03	17.4 ± 4.9
total (%)	5.7 ± 0.04	25.9 ± 4.0	94.7 ± 13.2

The relative abundance of the genera *Thauera*, *Paracoccus*, and *Azoarcus* in period 3 was higher than that in previous studies with an SRT of 10 days, with reported values of 84–88% and 83 ± 13%.^44,48,49^ The use of fermentation liquid rather than a synthetic carbon substrate greatly increased the relative abundance of these three genera in the SBR. This observation is consistent with the performance of the SBR described above in terms of *Y*_PHA/VFA_, −qVFA, and qPHA values. The presence of nutrients and a wider range of VFAs in the CPS compared to synthetic VFAs may improve biomass accumulation without compromising the selected microbial community. For example, period 3 was characterized by the presence of butyrate that may affect the growth of *Azoarcus* species in particular, resulting in a greater increase in abundance from period 2 to period 3 compared to the other two genera. Indeed, VFA composition is an important parameter affecting the microbial community in the S-SBR. *Azoarcus* and *Thauera* were previously shown to prefer acetate and butyrate, whereas *Paracoccus* spp. can grow on a broader range of substrates.^49^ Furthermore, *Thauera* was previously shown to be the dominant genus in the presence of acetate, whereas *Azoarcus* and *Paracoccus* were dominant in the presence of propionate.^53^ Our results confirmed that *Thauera* becomes the dominant genus when acetate is the main carbon source. The F/F value strongly influences the microbial population,^48^ with low F/F values favoring species that store the substrate rapidly because this offers a competitive advantage.^8,9^ The accumulation of PHA-storing bacteria during the experiment may reflect the corresponding decrease and stabilization of the F/F ratio from period 2 to period 3. Therefore, our results demonstrated that the operating conditions applied in the S-SBR achieved an optimal F/F ratio, driving the accumulation of PHA-storing bacteria.

Unlike the traditional aerobic F/F phases, we alternated between aerobic-feast and anoxic-famine phases to select for PHA-storing bacteria while abating the nitrogen content via the nitritation pathway. The greater efficiency of nitrite removal in period 3 may reflect the abundance of bacteria that utilize stored PHAs as a carbon source for denitrification. *Thauera* is a genus of denitrifying bacteria that can switch to denitrification and use nitrate, nitrite, or nitrogen monoxide as electron acceptors under low-oxygen conditions, and both *Paracoccus* and *Azoarcus* were previously isolated from the activated sludge of a denitrifying reactor.^54,55^ Furthermore, *Paracoccus denitrificans* is a nitrate-removing bacterium isolated from a fluidized-bed reactor and alternating anaerobic/aerobic and anaerobic/anoxic switch reactions.^56^ However, further research is needed to determine a cause–effect correlation between the SRT, F/F ratio, and microbial population selection.

## Conclusions

We have investigated for the first time the effect of the SRT and carbon source on the selection of PHA-storing biomass in terms of PHA production capability. We evaluated the changing microbial community during the experiments by PCR-DGGE and FISH to link the relative abundance of different bacteria with the operating conditions. We found that an SRT of 7–10 days rather than 5 days (period 1) conferred greater stability on the process, resulting in higher PHA production capacity. In period 2, the PHA production yield was 0.40 ± 0.05 gCOD_PHA_/gCOD_VFA_. We found that the highest PHA production yield of 0.62 ± 0.04 gCOD_PHA_/gCOD_VFA_ was achieved using fermented CPS rather than synthetic VFAs even though CPS contained non-VFA fractions that were not efficiently consumed under anoxic-famine conditions. Finally, the microbial community was strongly influenced by the SRT. The combination of an SRT of 7–10 days and fermented CPS as a carbon source resulted in the accumulation of three bacterial genera (*Thauera*, *Azoarcus*, and *Paracoccus*), representing ∼95% of the total biomass.

## Supporting Information Available

Description of the reactor configurations, table showing the sequencing of bacterial 16S rDNA bands excised from the DGGE gel, and FISH images stained with DAPI and specific probes for the genera *Thauera*, *Paracoccus*, and *Azoarcus* (PDF)
