# Supplementary material for: Effects of the Sludge Retention Time and Carbon Source on Polyhydroxyalkanoate-Storing Biomass Selection under Aerobic-Feast and Anoxic-Famine Conditions
Source: ACS Sustain Chem Eng. 2021 Jul 8;9(28):9455–64. doi: 10.1021/acssuschemeng.1c02973 (PMC8764655; doi:10.1021/acssuschemeng.1c02973)
Supplement: Supplementary file 1 — sc1c02973_si_001.pdf [file sc1c02973_si_001.pdf]

Supplementary materials for:

Effect of sludge retention time and carbon source on polyhydroxyalkanoate-storing biomass selection under aerobic-feast and anoxic-famine conditions

Nicola Frison[†,*], Marco Andreolli[†,*], Alice Botturi[†], Silvia Lampis[†], Francesco Fatone[‡]

† Department of Biotechnology, University of Verona. Strada Le Grazie 15, 37134 Verona, Italy.

‡ Department of Science and Engineering of Materials, Environment and Urban Planning-SIMAU, Marche Polytechnic University, via Brecce Bianche 12, 60131 Ancona, Italy

[*]corresponding authors

Nicola Frison: E-Mail, nicola.frison@univr.it; Tel. 0039 045 802 7965

Marco Andreolli: E-Mail, marco.andreolli@univr.it; Tel. 0039 045 802 7095

Alice Botturi: E-Mail, alice.botturi@univr.it;

Silvia Lampis: E-Mail, silvia.lampis@univr.it;

Francesco Fatone: E-Mail, f.fatone@staff.univpm.it

Number of pages: 7

Number of figure: 1

Number of tables: 3

Reactor configurations

Sequencing batch fermentation unit (SBFR). The SBFR, which treats cellulosic primary sludge (CPS) from sieved wastewater for the production of VFAs, has been previously described.[1] The SBFR had a working volume of 4 L and operated under stable mesophilic conditions (37 ± 1 °C). The hydraulic retention time (HRT) was controlled at 4 days. Every day, the effluent from the SBFR was harvested and centrifuged at 2100 × g to separate the solid and liquid fractions.

Nitritation SBR (N-SBR). The N-SBR was used for the via-nitrite treatment of raw anaerobic reject water produced by the anaerobic digestion of sewage, taken from the municipal waste water treatment plant in Carbonera (Treviso, Italy). We initially characterized the specific oxygen uptake rate (sOUR), specific ammonia uptake rate (sAUR) and specific nitrogen utilization rate (sNUR). The results (**Table S1**) were standardized by the MLVSS concentration and the reference temperature (20 °C).

Table S1. Specific nitrification and denitrification activity found in the inoculum.

Kinetics	Unit	Average	Min–Max
sOUR (20°C)	mgO2/gVSS h	24 ± 3	(19–35)
sAUR (20°C)	mgN/gVSS h	1.5 ± 0,2	(1.2–1.8)
sNUR (20°C)	mgN/gVSS h	2.1 ± 0,3	(1.6–2.6)

The working volume of the N-SBR was 15 L and it was equipped with an air blower with ceramic porous diffusers installed in the base of the reactor to achieve an oxygen concentration in the range 1.5–2.0 mg/L during the aerobic phases. The reactor was also equipped with an oxygen sensor (Hach-Lange, Düsseldorf, Germany) and RW 20 overhead stirrer (IKA-Werke, Staufen, Germany) with a blade impeller to maintain the activated sludge under agitation during the reaction phases. The anaerobic reject water contained ~1 g NH_4-N/L and the COD/NH_4-N was < 1. The main chemical characteristics of the anaerobic reject water are summarized in **Table S2**.

Table S2. Chemical characteristics of the anaerobic reject water.

Parameter	Unit	Value (average ± std)
NH_4-N	mgN/L	1060 ± 112
PO_4-P	mgP/L	27.27 ± 13.06
pH	-	7.66 ± 0.16
Total alkalinity 5.7	mg$CaCO_3$/L	4557.43 ± 975.6
Partial alkalinity 4.3	mg$CaCO_3$/L	5733.86 ± 1296.67

The sludge reject water was fed using a peristaltic pump and a flow rate of 21 L/h whereas the peristaltic pump used to discharge the nitrified supernatant had a flow rate of 4.2 L/h. The anaerobic reject water was fed with a vNLR of up to 1.6 kgN/m^3 day, which achieved an ammonia conversion efficiency of 85%, corresponding to an average concentration of nitrite in the effluent of 0.9–1.0 g NO_2-N/L. The nitritation reactor cycled through different phases governed by a programmable logic controller. The phases were: (1) feeding, 12 min; (2) aerobic phase, 180 min; (3) settling, 10 min; and (4) discharging, 20 min. A stable via-nitrite pathway from the oxidation of ammonia was completed in ~15 days. The stability was evaluated by calculating the ratio of the NO_2-N concentration in the effluent to the NO_3-N+NO_2-N concentration. The free ammonia content was maintained at 1–10 mg N/L to favor the ammonium-oxidizing bacteria (AOB) and limit the growth of nitrite oxidizing bacteria (NOB), and the pH was kept at 7.6–8.2. The alkalinity was also maintained by pH control (dosing ~6 g $CaCO_3$/L of rejected water). This strategy has been described in detail.[2] The effluent from the N-SBR was discharged to a 100-L storage tank and then used as source of electron acceptors for the S-SBR under anoxic-famine conditions.

Accumulation SBR (A-SBR). The A-SBR had a working volume of 2 L and was equipped with a magnetic stirrer (VELP Scientifica, Usmate Velate, Italy) to maintain complete agitation of the selected biomass, and oxygen sensors (Hach-Lange). Inoculum in the A-SBR was used to increase

the PHA concentration by the addition of VFAs under complete aerobic conditions. An air blower with ceramic porous diffusers provided oxygen to the biomass during the reaction. The VFAs were added based on demand as previously described.[2,3] Tests were conducted under aerobic conditions with excess carbon substrate, which was spiked to the biomass at a concentration of 1 gCOD VFA/L. The data obtained from the A-SBR were not reported because they were beyond the scope of our study.

Table S3. Sequencing analysis of bacterial 16S rDNA bands excised from the DGGE gel.

Band	Closest bacterial strain	Acc. No.	Percentage identity	Class
**S3**	Uncultured *Thauera* sp. clone 30-S4	JQ724357	100	β Proteobacteria
	Thauera butanivorans NBRC 103042(T)	BCUG01000113	96	β Proteobacteria
	Thauera linaloolentis 47Lol(T)	AMXE01000135	96	β Proteobacteria
**S4**	*Pseudomonas sediminis* PI11(T)	NIQU01000015	100	γ Proteobacteria
	Pseudomonas nitrititolerans GL14(T)	MH917718	100	γ Proteobacteria
**S5**	*Pseudomonas composti* C2(T)	FN429930	100	γ Proteobacteria
	Pseudomonas indoloxydans Bss-13a	KX161387	100	γ Proteobacteria
**S6**	Uncultured *Leadbetterella* sp. SSP-AOLR-2-1	KR705991	99	Cytophagia
	Flavobacterium sp. clone SA_NR2_1	GU726988	99	Flavobacteriia
**S9**	*Allorhizobium undicola* ATCC 700741(T)	JHXQ01000045	99	α Proteobacteria
	Devosia chinhatensis IPL18(T)	JZEY01000061	99	α Proteobacteria
**S13**	*Paracoccus aminovorans* isolate 35N_16392	LT690976	100	α Proteobacteria
**S16**	Uncultured *Thauera* sp. 20-NS11	JQ724337.1	100	β Proteobacteria

	Thauera butanivorans NBRC 103042(T)	NBRC 103042(T)	96	β Proteobacteria
**N5**	*Anaerobium acetethylicum* GluBS11	NR_137405	97	Firmicutes
	Uncultured *Lachnospiraceae* 16-bac-acetate	KT895571	97	Firmicutes
	Parasporobacterium paucivorans SYR1	NR_025390	96	Firmicutes
**N6**	Uncultured *Rhodopila* sp. CJ	FJ495222	99	α Proteobacteria
	Uncultured *Parvibaculum* sp. CH	FJ49522	99	α Proteobacteria
	Parvibaculum sedimenti HXT-9(T)	MN565579	99	α Proteobacteria
**N10**	*Riemerella* sp. BAB-5795	KX350185	99	Flavobacteria
	Bergeyella sp. S21.2	KT354265	99	Flavobacteria
	Chryseobacterium lacus YLOS41(T)	MG641896	99	Flavobacteria
**N17**	Uncultured *Thauera* sp. 20-NS15	JQ724338	99	β Proteobacteria
	Thauera butanivorans NBRC 103042(T)	BCUG01000113	98	β Proteobacteria
	Thauera linaloolentis 47Lol	NR_025283	98	β Proteobacteria
**N18**	Uncultured *Thauera* sp. 30-S4	JQ724357	100	β Proteobacteria
	Thauera butanivorans NBRC 103042(T)	BCUG01000113	96	β Proteobacteria
	Thauera linaloolentis 47Lol(T)	AMXE01000135	96	β Proteobacteria

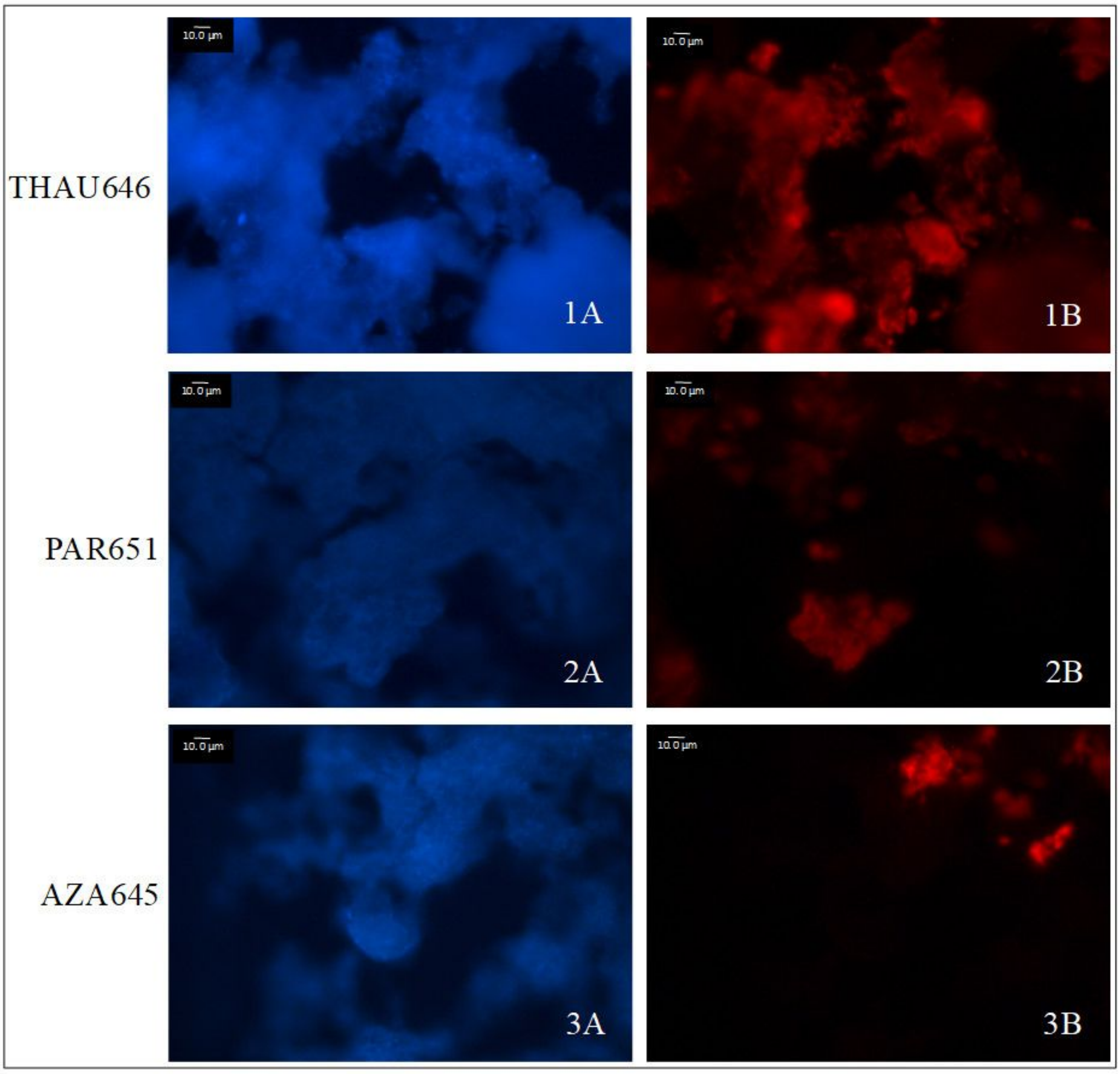

Figure S1. Representative FISH images (A) stained with DAPI and (B) with specific probes for the genera (1) *Thauera*, (2) *Paracoccus* and (3) *Azoarcus* on day 119. Bar = 10 µm.

References

(1) Crutchik, D.; Frison, N.; Eusebi, A. L.; Fatone, F. Biorefinery of cellulosic primary sludge towards targeted Short Chain Fatty Acids, phosphorus and methane recovery. *Water Res.* **2018**, *136*, 112-119.

(2) Conca, V.; da Ros, C.; Valentino, F.; Eusebi, A. L.; Frison, N.; Fatone, F. Long-term validation of polyhydroxyalkanoates production potential from the sidestream of municipal wastewater treatment plant at pilot scale. *Chem. Eng. J.* **2020**, *390*, 124627.

(3) Frison, N.; Katsou, E.; Malamis, S.; Oehmen, A.; Fatone, F. Development of a novel process integrating the treatment of sludge reject water and the production of polyhydroxyalkanoates (PHAs). *Environ. Sci. Technol.* **2015**, *49*(18), 10877-10885.
